# A Rare Periprosthetic Knee Joint Infection With *Clostridium perfringens*, the Result of 2-Stage Exchange Treatment: A Case Report and Review of Current Literature

**DOI:** 10.5435/JAAOSGlobal-D-22-00283

**Published:** 2023-07-18

**Authors:** Phillip Stokey, Tyler Canova, Hend Elsaghir, Maged Hanna

**Affiliations:** From The University of Toledo College of Medicine and Life Sciences Orthopaedic Center, Toledo, OH.

## Abstract

Prosthetic joint infections (PJIs) commonly result from aerobic gram-positive organisms and can lead to detrimental outcomes. However, it is rare for *Clostridium perfringens* to cause a PJI. Owing to its rarity, current literature lacks a comprehensive guide for the proper management of these PJIs. We report on the case of an 80-year old man who presented to our institution with concerns for sepsis secondary to a PJI with C. *perfringens* 25 years status post total knee arthroplasty. The patient was managed with two-stage revision and exchange. After stage one, the patient developed cholecystitis, which has been reported in prior cases of PJI due to C. *perfringens*. After concerns for sepsis had resolved and stage 1 was complete, the patient was managed with 6 weeks of IV antibiotics. Treatment was directed at gram-positives with IV vancomycin along with anerobic coverage determined by anerobic susceptibility testing. After the second stage, the patient was discharged with 3 months of oral antibiotic therapy. At the final 1-year follow-up, the patient was doing well without residual infection. This report reviews previous evidence on the management of C. *perfringens* PJI and presents a case demonstrating the successful diagnostic, surgical, and antimicrobial management of a PJI with C. *perfringens*.

Prosthetic joint infections (PJIs) commonly result from aerobic gram-positive organisms, with *Staphylococcal aureus* and *epidermidis* being the most likely culprits.^1^ These pathogens inoculate themselves during surgery, spreading hematogenously or from adjacent tissue. However, it is rare for Clostridium *perfringens* to cause infection of a prosthetic joint.^2^

Clostridium *perfringens* is an α-toxin–producing, spore-forming, obligate anaerobe, which appears as gram-positive rods on microscopy. These bacteria typically reside in the gastrointestinal tract and are known to cause food poisoning. When inoculated from the environment, these can cause a highly lethal myonecrosis termed gas gangrene. This historically occurred in war-related trauma injuries, preferentially infects hypoxic tissue, and produces a foul-smelling gas with crepitus under the skin.^3^

Despite derivation from the same causative organism, a PJI due to C. *perfringens* will present differently than a patient with gas gangrene. Instead, it will resemble an infection from gram-positive cocci with pain and effusion surrounding the joint, elevated inflammatory markers, and a turbid joint aspiration with leukocytosis and neutrophilia. However, in contrast to an infection due to gram-positive cocci, a PJI due to C. *perfringens* is less likely to present in the perioperative period because these bacteria typically lack the ability to colonize equipment or spread from adjacent tissue.^2^ Instead, they commonly seed equipment hematogenously months to years after placement.

An 80-year-old man presented to our institution with concerns for sepsis secondary to a PJI of the left knee with C. *perfringens*. A literature review was conducted to outline the proper management of this patient only to find it lacking a comprehensive guide. In fact, there have only been 10 other cases of Clostridium *perfringens* PJI reported in the literature previously.^2,4,5,6,7,8,9,10,11^ Of these cases, five involved hip prostheses and five involved knee prostheses.^4,5,6,7,8,9,10,11^ Only three of these cases exemplified a clear source of infection, and the infection arose from the biliary system in all three.^2,5,7,9,10^ We review previously published literature involving PJI due to C. *perfringens* (Table 1). Then, we report on a case. We highlight the successful diagnostic, surgical, and antimicrobial management of this patient, which has been supported both in this case and previously published articles in the hopes that this article serves as a comprehensive guide to orthopaedic surgeons with managing patients with PJIs due to C. *perfringens*.

**Table 1:** Summarization of Available Literature Found on Prosthetic Joint Infections due to C. Perfringens, Including Author, Year, Joint, Surgical Treatment, Infection Source, Antibiotics Management, and Outcome

Report Author (yr)	Joint	Surgical Treatment	Source	Antimicrobial management	Outcome
Rush (1976)	Hip prosthesis (2 cases)	—	—	—	—
Maniloff et. al (1987)	Hip prosthesis	—	—	—	—
Stern et. al (1988)	Knee Arthroplasty	—	—	—	—
Wilde et. al (1988)	Knee Arthroplasty	Removal, débridement, and arthrodesis	Acute cholecystitis	—	—
Vogely et. al (1999)	Right total hip prosthesis	2-stage revision	Acute cholecystitis	IV penicillin and oral clindamycin (time line not specified)	No recurrence at 1.5 years
Pearle et. al (2003)	Total Knee Arthroplasty	Irrigation, débridement, and removal and implantation of an antibiotic-impregnated cement spacer	—	—	—
Kibbler et. al (2005)	Hip Prosthesis	None	None identified	IV benzyl penicillin and Fucidin, followed by oral amoxicillin	No recurrence at 19 months
Zafar et. al (2019)	Left Knee Arthroplasty	Irrigation and débridement with polyethylene exchange without replacement of the prosthesis	Acalculous cholecystitis	6 weeks of IV ertapenem and 12 months of oral moxifloxacin	No recurrence at 6 months
Stroud et. al (2020)	Right Knee Arthroplasty	2-stage revision	None identified	IV clindamycin 900 mg TID, oral metronidazole 500 mg TID for weeks between revisions, and oral penicillin 500 mg QID for 1 year	No recurrence at publication

## Case Description

An 80-year-old man with a history of left primary total knee arthroplasty (TKA) 25 years ago presented with a 6-day history of left knee pain and swelling. The patient's medical history included obesity, chronic anemia, hypertension, and gout. No history of joint infection or surgery was reported since primary arthroplasty. An outside orthopaedic surgeon conducted an aspiration that grew C. *perfringens*; the patient was started on vancomycin, but because of concerns for sepsis, he was transferred to our facility.

**Figure 1:**
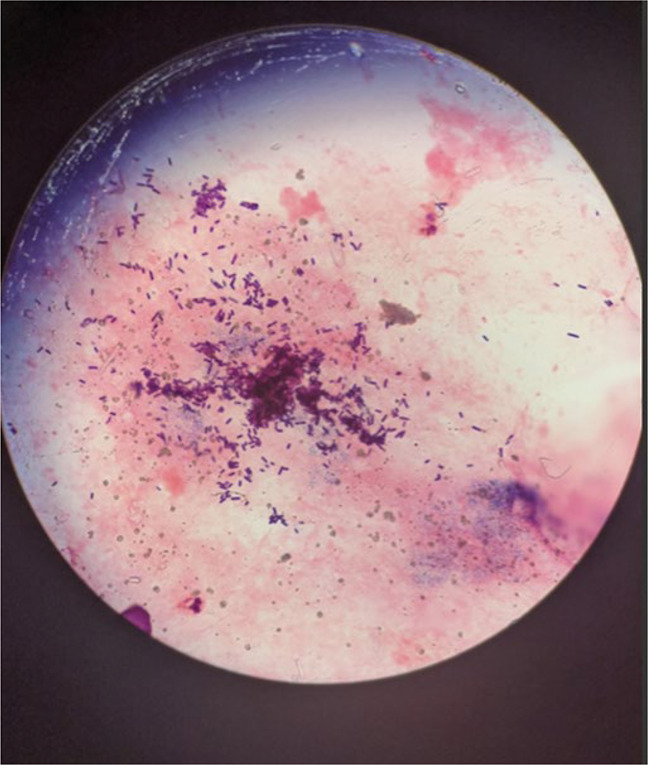
Image obtained during light microscopy imaging of Clostridium perfringens in culture.

**Figure 2:**
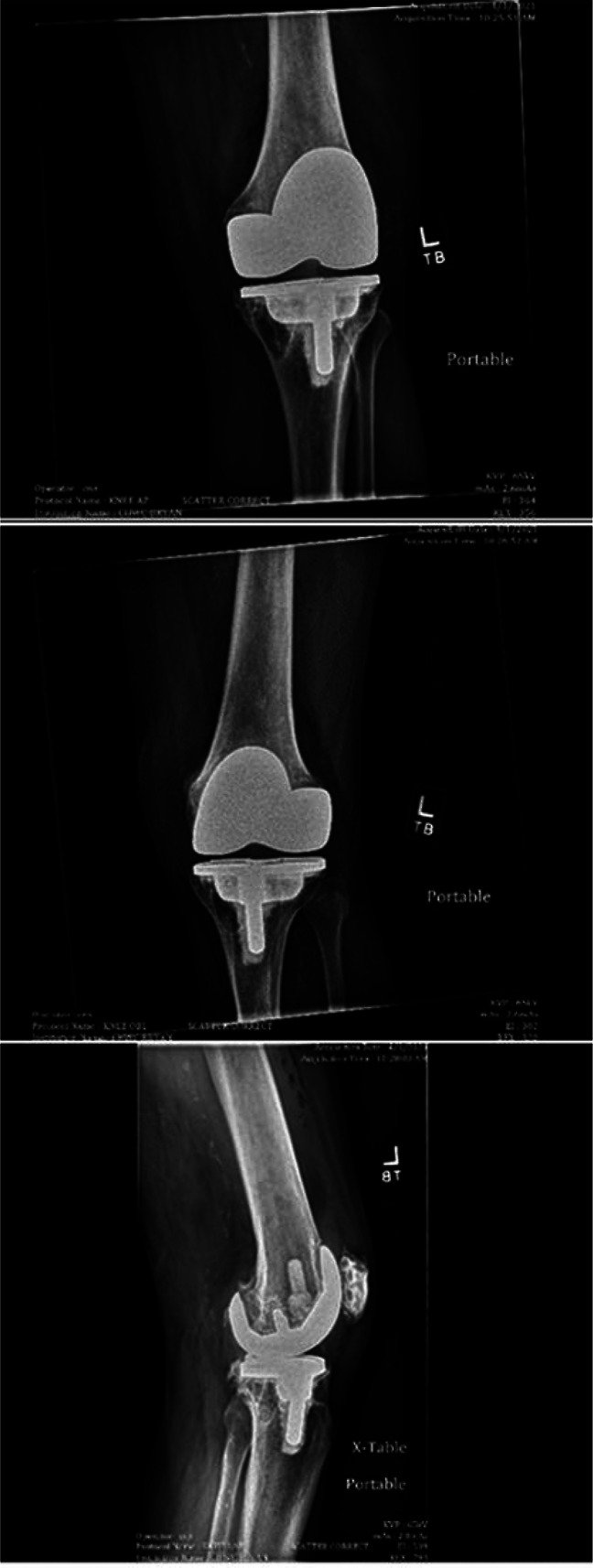
**A,** Anteroposterior (AP), (**B**) oblique, and (**C**) lateral radiographic views of left total knee arthroplasty at initial presentation showing soft-tissue swelling.

On presentation to us, his left knee demonstrated erythema and swelling. The vitals at presentation were stable, and laboratory values showed elevated white blood cells with notable neutrophilia (11.7 × 10^3^ WBCs/uL with 91% neutrophils) and markedly elevated inflammatory markers (CRP 284 mg/L and ESR 113 mm/hr). The patient's left knee was reaspirated at our hospital and yielded a purulent fluid. Microscopic examination of the aspirate revealed 30,000 white blood cells with 93% segmented neutrophils, and Gram staining of the fluid identified Clostridium *perfringens* (Figure 1). Radiograph of the left knee showed the presence of a TKA with evidence of osteolysis about the medial tibial baseplate with significant soft-tissue swelling (Figure 2). No advanced imaging was completed. The patient developed vancomycin-induced AKI and was transitioned to IV clindamycin and piperacillin-tazobactam.

**Figure 3:**
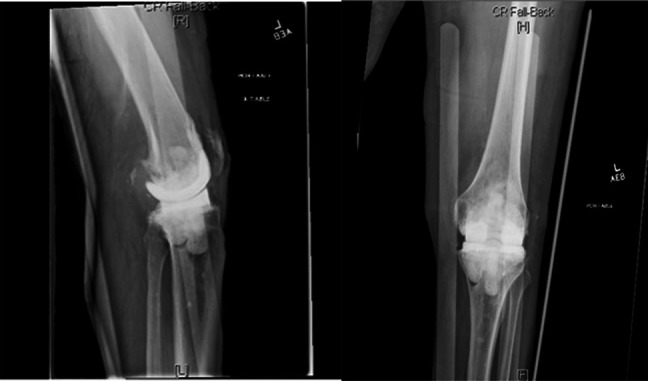
**A, B,** Postoperative radiographs with placement of the antibiotic spacer exemplifying proper anatomic alignment and no evidence for acute complication.

Given the aspiration results and clinical signs, the decision was made for explantation and stage I revision with the use of 1 gm of vancomycin and 3.6 gm of tobramycin added to each pack of cement and imbedded in the cement spacer, which proceeded without complications. The patient was started postoperatively on IV vancomycin, cefazolin, and metronidazole. Postoperative radiographs were obtained to confirm appropriate implant placement (Figure 3, A,B).

**Figure 4:**
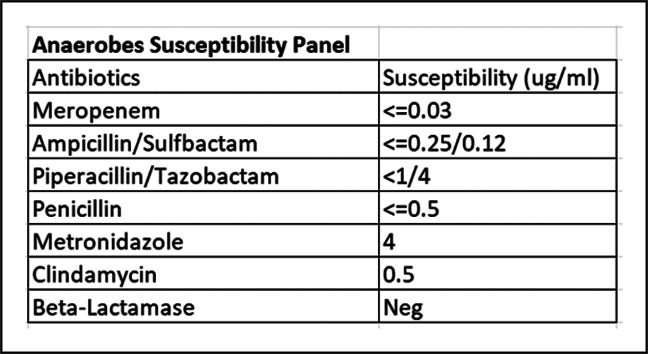
Diagram showing the antibiotic susceptibility panel from our case of Clostridium perfringens used to guide outpatient antibiotic management.

Postoperatively, the patient developed acalculous cholecystitis with elevated liver enzymes, which trended downward before discharge. Intraoperative cultures grew *Clostridium perfringens* in an anerobic culture. Complete intraoperative culture results are provided in Table 2. Susceptibility testing was completed and guided outpatient antibiotic management (Figure 4). From this, the infectious disease team recommended the continuation of antimicrobial therapy using a peripherally inserted central catheter. The patient was discharged with oral metronidazole q8 and IV ceftriaxone q24 hours for 6 weeks.

**Table 2:** Results of Intraoperative Cultures

Results of Intraoperative Cultures			
Tissue	Fungal culture	Aerobic	Anerobic
Left Knee Synovium #1	Culture-negative	Many Polys No bacteria seen No growth on day 5	No Anaerobes Isolated 5 days
Left Knee Synovium #2	Culture-negative	Many Polys No bacteria seen No growth on day 5	*Clostridium perfringens*
Left Knee Deep Synovium	Culture-negative	Many Polys No bacteria seen No growth on day 5	No Anaerobes Isolated 5 days
Left Knee Tibia Swab	Culture-negative	Many Polys Moderate Gram-Bacilli No growth on day 5	No Anaerobes Isolated 5 days
Left Knee Tibial Tissue	Culture-negative	Many Polys Moderate Gram-Bacilli No growth on day 5	Clostridium *perfringens*

After the patient finished 6 weeks of antibiotics, his inflammatory markers trended downward (CRP 18.2 mg/L, ESR 19 mm/HR), he was given 3 weeks of antibiotics holiday, and he was reaspirated in clinic to rule out infection. This aspiration, completed 9 weeks after stage 1, demonstrated 1921 WBCs/uL with 74% neutrophils. The patient was then scheduled for second-stage revision 5 months after the first stage.

**Figure 5:**
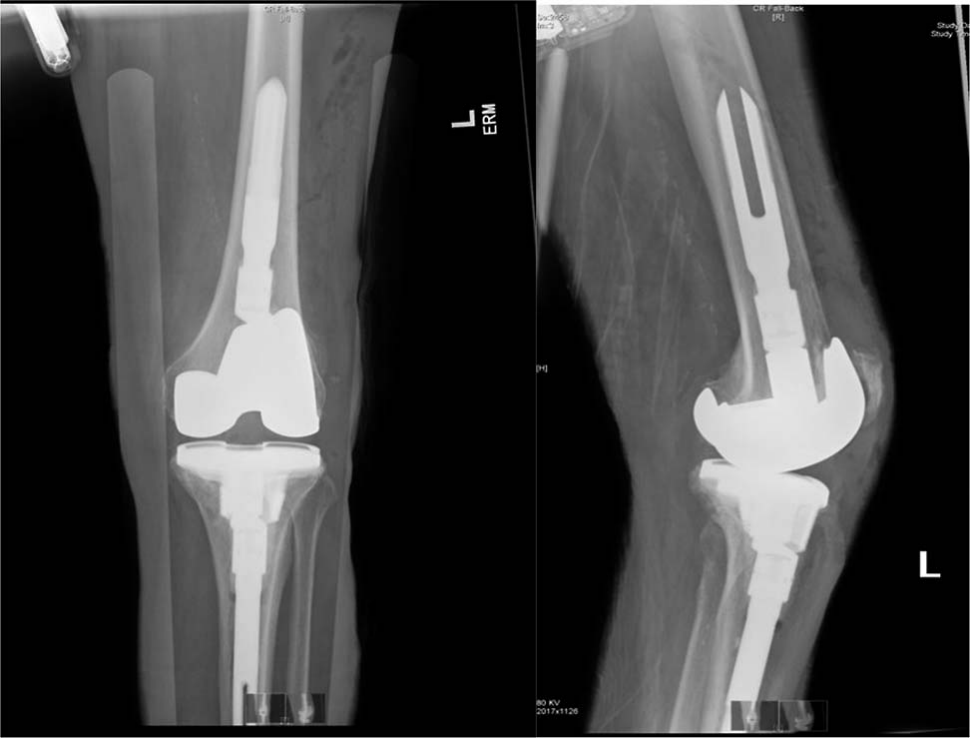
Radiographs demonstrating replacement of the long tibial and femoral stems after stage 2 of the revision. Alignment appears excellent. Air in the soft tissues is noted as expected.

In surgery, the knee was opened with no signs of infection or purulence and intraoperative frozen sections were obtained and sent to pathology, returning with <1 neutrophil/high power field, which indicates lower risk of persistent infection. Intraoperative cultures were obtained and sent for aerobic, anerobic, and fungal cultures, which returned negative. We decided to reimplant a revision prosthesis; stemmed femoral and tibial implants were used, as well as a tibial cone to achieve good zonal fixation (Figure 5).

After the procedure, the patient was started on a 3-month regimen of oral suppressive therapy with amoxicillin-clavulanate guided by the 2020 Mark Coventry trial.^12^ At the final follow-up status post stage 2 revision, the patient had no reports of joint instability, pain, or symptoms of recurrent infection.

## Discussion

Clostridium *perfringens* is an α-toxin–producing, spore-forming, obligate anaerobe, which appears as gram-positive rods on microscopy. Although these bacteria do not exhibit extensive antimicrobial resistance like those of S. *aureus*, they do produce an arsenal of virulent toxins capable of causing rapid myonecrosis and sepsis.^13^ In addition, they attain the ability to exist as dormant spores. Together, these factors pose challenges in effectively managing these infections.

On presentation, the patient in our case did not meet SIRS criteria, but demonstrated a positive joint aspiration.^14^ Because the patient underwent TKA over 25 years ago, equipment colonization was unlikely. There is the possibility of C. *perfringens* as a contaminant, but two positive joint aspirations with positive intraoperative cultures make the same unlikely. Our patient experienced cholecystitis after explantation of the joint. This, combined with evidence citing PJIs with Clostridium *perfringens* secondary to cholelithiasis, raises suspicion to the biliary system as a source of the original infection.^2,5,7,9,10^

If the biliary tract was the source of infection, it is necessary to review the factors that caused us to fail to consider this earlier. One scenario points to the presence of asymptomatic gallstones allowing for the initial growth of *Clostridium perfringens*, which led to hematogenous seeding of the joint. To our knowledge, the case reported by Zafar et al^2^ was the only one that replicated the sequence of symptom presentation seen in our patient. In our report, the patient presented with sepsis secondary to a prosthetic joint aspirate that grew *Clostridium perfringens*. It was later found that the patient had acalculous cholecystitis that allowed for the initial growth. Wilde et al^7^ also reported on a patient who presented with sepsis after TKA, but the cholelithiasis became apparent before joint explantation. Earlier recognition of the source of infection may lead to altered treatment plans and improve outcomes.

Two-stage arthroplasty exchange was completed in three of the 10 cases reported on *Clostridium perfringens* PJI.^2,4,5,6,7,8,9,10,11^ Zafar et al reported the successful débridement of a knee PJI without prosthesis removal, and Kibbler et al reported on successful antibiotic treatment alone without surgical intervention^2,6^ (Table 1). In this case, the timing of infection (25 years status post primary equipment implantation), infection progression, the limited evidence of C. *perfringens* PJI, and the knowledge that these bacteria were capable to exist as inactive spores contributed to the decision to undergo two-stage revision instead of one-stage revision.

This case highlights a mode of antimicrobial therapy that was successful in eradicating a PJI with C. *perfringens*. After identification of the pathogen as *clostridium*, most evidence supports using penicillin or vancomycin in the preoperative period.^2,6-8,10^ This case is unique, in that vancomycin had to be discontinued because of concerns for AKI. Instead, clindamycin and piperacillin/tazobactam were initiated. Clindamycin, in particular, may be especially helpful in the acute phase because it suppresses the alpha-toxin activity of *Clostridium*.^10,15^ After Stage 1 revision, a dual combination antibiotic regimen should be initiated for 6 weeks. Vancomycin is the evidence-based choice for outpatient gram-positive coverage. However, as demonstrated here, the proper secondary antimicrobial agent should target anaerobes and susceptibility testing should guide this choice. After two-stage revision, a 3-month regimen of oral antibiotics should be initiated to reduce the rate of failure.^12^

## Conclusion

When PJI by Clostridium *perfringens* is confirmed, suspicion should lead toward the biliary system as a source. After stage 1 revision, a 6-week antimicrobial regimen should be initiated using vancomycin and a second agent with anerobic coverage determined by susceptibility testing. After stage 2, the patient should be placed on 3 months of oral antimicrobials.
